# Sepsis as a Challenge for Personalized Medicine

**DOI:** 10.3390/jpm12121989

**Published:** 2022-12-01

**Authors:** Roman Zahorec, Miroslav Průcha

**Affiliations:** 1 Department of Anesthesiology and Intensive Medicine, Medical School, Comenius University, Heydukova 10, 812 50 Bratislava, Slovakia; 2 Department of Clinical Biochemistry, Haematology and Immunology, Na Homolce Hospital, 140 00 Prague, Czech Republic

Sepsis is a clinical syndrome of systemic inflammation induced by infection, now defined as life-threatening organ dysfunction caused by a dysregulated immune response to infection [1]. A dysregulated host response means an acute imbalance between invading microorganisms and the innate immune system. In other words, sepsis involves acute failure of the innate immune system against invading microbes. Thirty years ago, when Roger Bone established the concept of Sepsis and SIRS based on SIRS criteria, thousands of articles were issued on this topic. The remarkable progress in clinical medicine in the field of Sepsis can be attributed to basic research, genomics and proteomics, together with a better understanding of the immunopathology, biology and epidemiology of sepsis syndrome. The aim of this Special Issue is to provide research evidence and potential uses for personalized medicine in Sepsis, highlighting eight papers focused on research achievements in animal and human studies. Experimental models of sepsis can provide a clear understanding the pathophysiology of sepsis and its evolution to septic shock. Czech authors studied the early phase of sepsis on a porcine model of peritonitis-induced systemic infection. They followed up on cardiovascular deterioration, and hemodynamic and biochemical parameters in the first 24 h of sepsis. Animals (pigs) who developed septic shock had a higher heart rate, significantly higher cardiac output and a SOFA score underlying a higher concentration of interleukin IL-6, TNF-alfa and lactate. Pigs who developed septic shock had hyperpyrexia over 40 degrees Celsius when compared with those with mild sepsis (median around 39 °C). The authors observed remarkable differences in organ dysfunction according to the SOFA scores at the end of the study period, 24 h after developing sepsis [2]. Proteomics technologies have potential in the early diagnosis and treatment of sepsis. Physicians from Taipei Medical University explored the effect of the novel peptide KCF18 on the course of endotoxemia in mice. The peptide KCF18 can modulate the course of sepsis and alleviate organ injury (liver) via the simultaneous inhibition of three mayor cytokines (TNF-alfa, IL-1beta and IL-6) [3]. Effective therapy relies on the early and precise diagnosis of sepsis. Personalized medicine needs a reliable diagnostic algorithm(s) to achieve fast microbiology and identify biomarkers of infection and inflammation for the appropriate treatment of sepsis. Valuable data on the role of proadrenomedullin in the accurate identification of H1N1 influenza in viral sepsis was provided in an article by a Spanish research group [4]. Severe viral pneumonia induced by influenza virus A (H1N1) may lead to acute respiratory failure and the need for mechanical ventilation with high risk of death. The study compared the effectiveness of mid-regional proadrenomedullin (MR-proADM) to the C-reactive protein, procalcitonin and ferritin for anticipating ICU and 90-day mortality. Serum concentrations (cut-off values) of MR-proADM (higher than 1.2 mmol), ferritin, CRP and procalcitonin (higher than 2.75 µg/L) effectively determined adverse outcomes and mortality in patients with H1N1 influenza viral pneumonia [4]. MR-proADM may be a reliable marker in the decision-making process in emergency departments because its values correlate with illness severity, and the need for hospital treatment (values higher than 0.87 nmol/L) and ICU treatment (higher than 1.0 nmol(L), with significant incidence of mortality when values are greater than 1.5 nmol/L [5,6]. Myristic acid, a new metabolic biomarker of SIRS, sepsis and bacteremia, is released into the blood very early after the onset of infection with bacteremia and sepsis. Zazula et al. [7] demonstrated dynamic changes in the serum concentration of myristic acid during bacteremia earlier than procalcitonin. Myristic acid is quite a new perspective inflammation marker of lipid origin, and has been studied extensively by other authors researching the metabolomics of sepsis. Myristic acid was the single most predictive metabolite with highest sensitivity and specificity for bacteremia sepsis in an emergency department [8,9]. Among the standard biomarkers of sepsis, presepsin seems to be a valid parameter for identifying patients with infection and sepsis. A single-center prospective study performed at Craiova Emergency Hospital explored the role of presepsin on a cohort of 114 adult patients admitted to the ICU. Presepsin serum concentrations were associated with patients with sepsis (median 1476 ng/mL) and septic shock (median 2403 ng/mL) and had the ability to differentiate between survivors and non-survivors. The high quality of presepsin was confirmed by a strong correlation with the SOFA score [10]. An interesting issue is that of the phenotype of sepsis based on the transcriptomic host response, which is divided into three endotypes—inflammatory (IE), coagulopathic (CE) and adaptive (AE) [11]. The authors performed a retrospective analysis in a subgroup of patients with sepsis treated with ascorbic acid, thiamine and glucocorticoids. In another study, a pre-established 33-mRNA classifier determined three sepsis endotypes [12]. The highest mortality was observed in the coagulopathic endotype (40%) followed by the inflammatory endotype (10%) and lowest was observed in AE (5%). The limitations of the study were its low number of participants and the fact that in CE patients, no data were collected on D-dimers, fibrinogen levels and thromboelastography. These limitations decreased the validity of the study [11]. The aim of the final manuscript, regarding individualized hemodynamic management in sepsis, was to give a pathophysiological rationale for the clinical application of modern approaches in the hemodynamic management of septic patients, applying multimodal and personalized management [13]. With sepsis research proving an ongoing challenge, this Special Issue aims to provide evidence for traditional clinical approaches to diagnosing and treating sepsis; moreover, it aims to highlight personalized medicine perspectives regarding the phenotypes and endotypes of sepsis, new emerging biomarkers and possible new treatment modalities.
